# Visible-light-mediated copper photocatalysis for organic syntheses

**DOI:** 10.3762/bjoc.17.169

**Published:** 2021-10-12

**Authors:** Yajing Zhang, Qian Wang, Zongsheng Yan, Donglai Ma, Yuguang Zheng

**Affiliations:** 1 Traditional Chinese Medicine Processing Technology Innovation Center of Hebei Province, Hebei University of Chinese Medicine, Shijiazhuang, 050200, P. R. China

**Keywords:** copper-photocatalyzed reactions, green chemistry, mechanisms of copper photocatalysis, photoinduced copper-based catalysis, photoredox catalysis, special features of copper photocatalysis

## Abstract

Photoredox catalysis has been applied to renewable energy and green chemistry for many years. Ruthenium and iridium, which can be used as photoredox catalysts, are expensive and scarce in nature. Thus, the further development of catalysts based on these transition metals is discouraged. Alternative photocatalysts based on copper complexes are widely investigated, because they are abundant and less expensive. This review discusses the scope and application of photoinduced copper-based catalysis along with recent progress in this field. The special features and mechanisms of copper photocatalysis and highlights of the applications of the copper complexes to photocatalysis are reported. Copper-photocatalyzed reactions, including alkene and alkyne functionalization, organic halide functionalization, and alkyl C–H functionalization that have been reported over the past 5 years, are included.

## Introduction

Solar light is an inexhaustible and free energy source for green plants and bacteria. Photosynthetic organisms absorb solar energy and convert it into chemical energy via photosynthesis [1]. Photochemical reactions mimic natural photosynthesis, and photoredox catalysis plays a key role in energy-transfer processes [2–5]. Over the past decades, photoredox catalysis has attracted an increasing amount of attention [6–9], and a series of organic dyes and metal complexes have been investigated [10–12]. Photoredox catalysts have been initially applied to organic reactions, but they are now used for complicated organic processes [13]. As photocatalysts, organic dyes have the advantages of having a low price and not containing metals; however, they suffer from relatively poor photostability [14–16]. Transition-metal-photoredox catalysts, such as ruthenium and iridium polypyridyl complexes, exhibit high redox potentials, long excited state lifetimes, and strong absorption [17–20]. However, high cost and their scarcity discourage development of ruthenium and iridium-based catalysts [21]. Copper salts have become popular materials for photoredox catalysts due to their abundance, low cost, and ability to provide strong photoexcited reducing power [21–24]. In this review, the different catalysis mechanisms between ruthenium-based catalysts and copper-based catalysts are discussed, and the strong reduction ability of copper complexes is explained. Subsequently, mechanisms of the photoredox catalysis by Cu^I^ and Cu^II^ are summarized, and the copper-catalyzed reactions, including alkene functionalization, alkyne functionalization, organic halides functionalization, and alkyl C–H functionalization, are highlighted.

## Review

### 1. Special features of photoredox-catalyzed processes by copper complexes

**Scheme 1:**
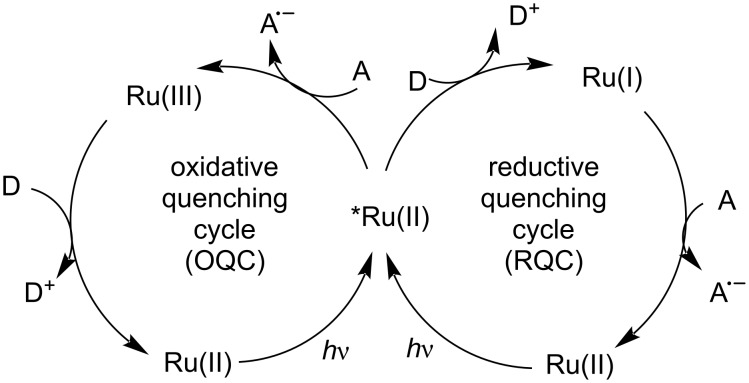
Photoredox catalysis mechanism of [Ru(bpy)_3_]^2+^.

To understand photoredox-catalyzed processes, a discussion of the general mechanism of [Ru(bpy)_3_]^2+^ is needed [25–27]. When the photocatalyst Ru^II^ is irradiated by light, an electron is transferred from the frontier metal d orbital (t_2g_ orbital) to the ligand-centered π* orbital (Ru^II^*). A metal-to-ligand charge transfer (MLCT) results in the excited singlet state. Through rapid intersystem crossing (ISC), the singlet state is transformed to the lowest-energy triplet MLCT state, which has a sufficient lifetime for initiating single-electron transfer. In the triplet species, the electron in the higher singly occupied molecular orbital (SOMO) is transferred from Ru^II^* to an external acceptor (A), thereby yielding oxidized Ru^III^, which subsequently accepts an electron from an external donor (D) to form the ground-state catalyst Ru^II^. This type of reaction mechanism is an oxidative quenching cycle (OQC). Alternatively, the lower energy SOMO of the excited state Ru^II^* can accept an electron from an external donor, which is referred to as a reductive quenching cycle (RQC; Scheme 1).

**Scheme 2:**
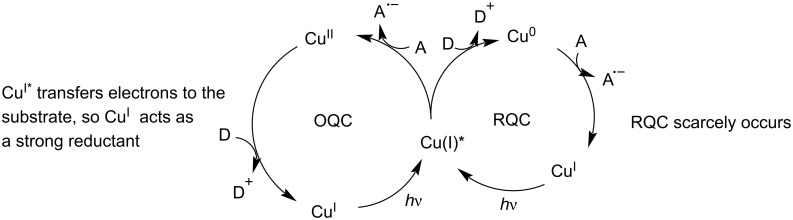
Photoredox catalysis mechanism of Cu^I^.

Compared with the photoredox mechanism of ruthenium-based catalysts, copper complexes show unique features [22,28]. Under irradiation, the copper complex Cu^I^ is converted to the excited state Cu^I^*, which transfers electrons to an acceptor A or receives electrons from a donor D. In the OQC pathway, the excited state Cu^I^* transfers an electron to the acceptor A and is oxidized to Cu^II^. Subsequently, Cu^II^ accepts an electron from the donor D to form the ground-state Cu^I^ (Scheme 2). However, reports that the excited state Cu^I^* receives electrons from donors are relatively scarce in the literature. Thus, the RQC pathway rarely occurs for Cu^I^-photocatalyzed reactions. Yet, Cu^I^ complexes have the potential to replace ruthenium or iridium-based photocatalysts in reductive photoredox reactions due to their strong reduction ability [22,29]. For example, [Cu(dap)_2_]Cl (*Cu^+^/Cu^2+^ = −1.43 V) provides a stronger reducing power than [Ru(bpy)_3_]Cl (*Ru^2+^/Ru^3+^ = −0.81 V) and [Ir{dF(CF_3_)ppy}_2_(dtbbpy)]Cl (*Ir^2+^/Ir^3+^ = −0.89 V) [28,30]. Nevertheless, upon absorbing a photon, Cu^I^ undergoes a reorganization from a tetrahedral geometry to a square-planar geometry, thereby resulting in a shorter excited state lifetime compared with ruthenium and iridium-based photocatalysts and thus limiting the application of Cu^I^ complexes to visible-light-mediated organic syntheses [22,31].

**Scheme 3:**
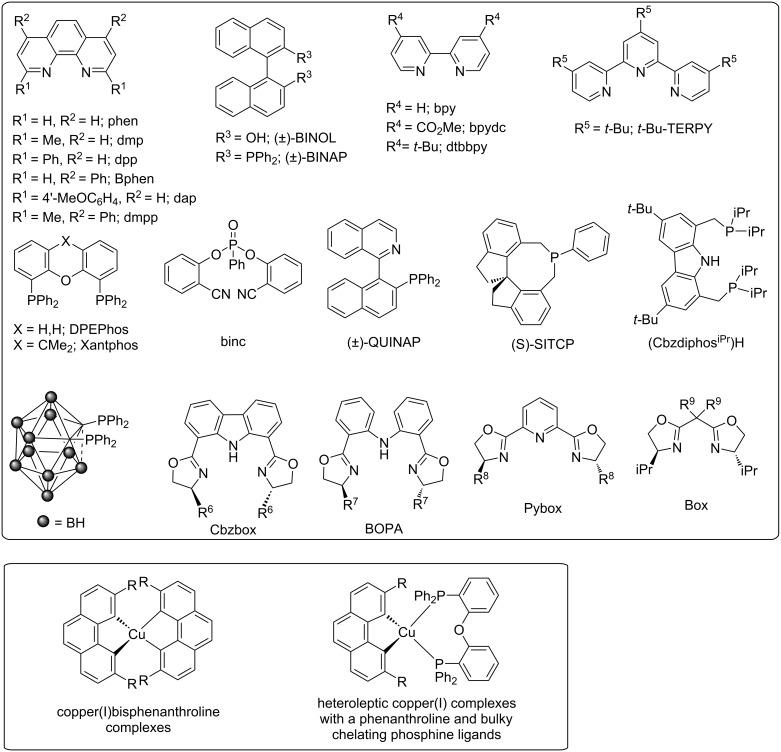
Ligands and Cu^I^ complexes.

Homoleptic Cu^I^ bisphenanthroline complexes were designed with cooperative steric hindrance based on bulky substituents at the 2,9-position of the phenanthroline moiety [32–33]. Alternatively, heteroleptic Cu^I^ complexes with phenanthroline and bulky chelating phosphine ligands were also synthesized [30,34–35]. The photophysical properties are dramatically modified by the homoleptic and heteroleptic Cu^I^ complexes [22,31,36]. The introduction of bulky ligand substituents might efficiently prevent the reorganization of the excited state. Thus, changing the nature of the chelating ligand can improve the photostability and lifetime of the excited state to meet the requirements of a given photochemical process. The different ligands and Cu^I^ complexes are shown in Scheme 3 [21,30]. The catalysis mechanisms of these Cu^I^ complexes are discussed in the following sections.

### 2. Mechanisms underlying the photoredox catalysis of copper complexes

The mechanisms underlying the photoredox catalysis of Cu^I^ complexes with different ligands were investigated by Reiser’s group [21]. Other studies have since provided more information on the photoredox mechanisms underlying the catalysis of copper complexes [37]. In general, redox-active copper complexes include Cu^I^ and Cu^II^ complexes. The mechanisms underlying photoredox catalysis of Cu^I^ complexes have special features and include ligand exchange and rebound mechanisms [38]. Cu^II^ complexes provide new avenues for photoredox catalysis, since Cu^II^ can undergo ligand exchange/light accelerated homolysis processes, which accelerates homolysis to produce Cu^I^ species and radical intermediates. These intermediates can initiate productive organic transformations [39].

#### 2.1 Visible-light-mediated Cu(I) catalytic cycle

**Scheme 4:**
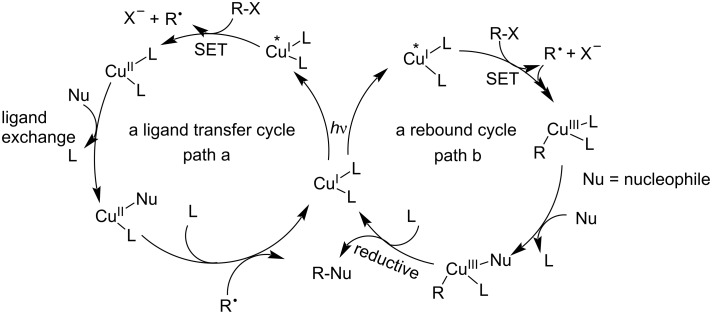
Mechanism of Cu^I^-based photocatalysis.

Upon the absorption of a photon (Scheme 4), Cu^I^L_n_ forms a singlet MLCT state, which subsequently yields the excited triplet state Cu^I*^L_n_ via rapid ISC. The excited Cu^I*^L_n_ species has a lifetime to finish the chemical processes. A radical mechanism is proposed in Scheme 4. In path a (a ligand transfer cycle), Cu^I^* is oxidized by an electrophilic reagent (haloalkane) to form Cu^II^ and the radical species R^•^ in a single-electron transfer (SET) process. Subsequently, Cu^II^ undergoes ligand exchange with a nucleophilic reagent (Nu) to produce the Cu^II^−Nu species. The reorganization of Cu^II^–Nu is trapped by the radical intermediate R^•^ to generate the final product (R–Nu) with concomitant regeneration of the Cu^I^ catalyst. Alternatively, in path b (a rebound cycle), Cu^I*^ is trapped after a SET by the radical intermediate to generate a Cu^III^ species, which undergoes ligand exchange with the nucleophile and reductive elimination to produce the target product and the regenerated Cu^I^ catalyst [37–38,40].

#### 2.2 Visible-light-mediated Cu(I)–substrate catalytic cycle

**Scheme 5:**
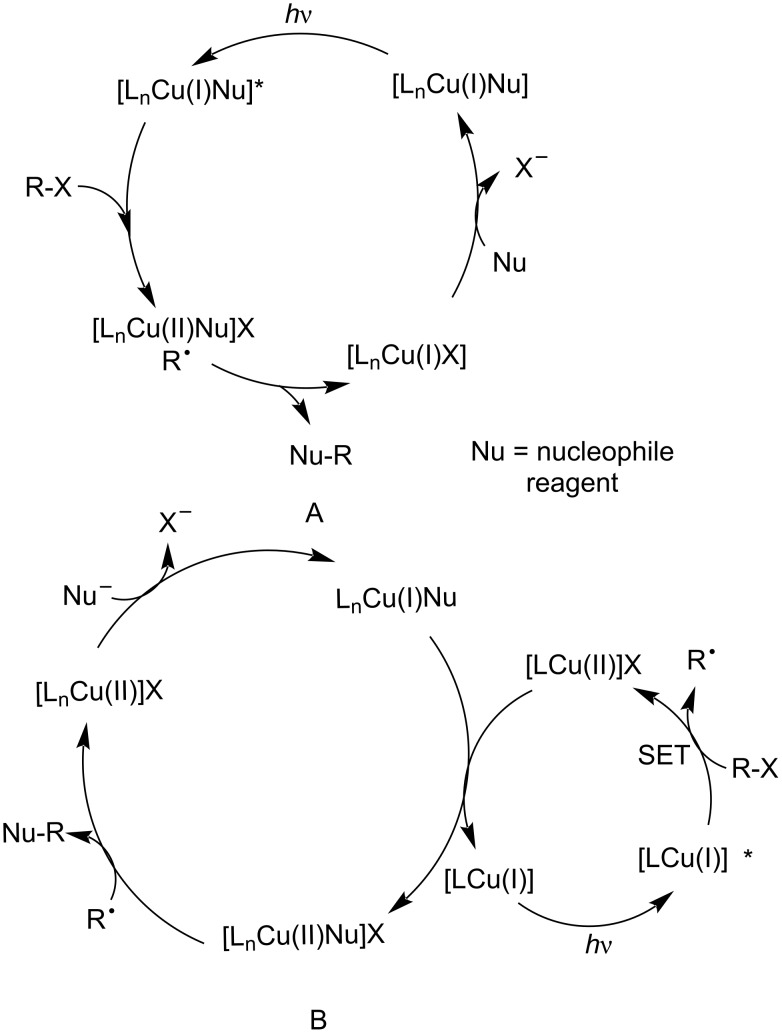
Mechanisms of Cu^I^–substrate complexes.

Upon the irradiation of L_n_Cu^I^Nu, an electron is transferred from the metal center to the ligand, thereby generating the excited state L_n_Cu^I^Nu^*^. The excited state species can be oxidized by the electrophilic reagent (haloalkane, RX) to form the intermediate [L_n_Cu^II^Nu]X. The desired product Nu–R can be obtained through an inner-sphere pathway between [L_n_Cu^II^Nu]X and the radical R^•^ [41–42] (Scheme 5A). Alternatively, a photosensitizer generated a radical via reduction or oxidation, and is not engaged in the key bond construction. [LCu^I^] is photoexcited to generate L_n_Cu^I^*, which transfers an electron to the haloalkane, thereby resulting in the formation of [LCu^II^]X and R^•^. Then, the radical R^•^ is trapped by a second copper complex [L_n_Cu^II^Nu]X, which mediates Nu–R bond formation in an out-of-cage process (Scheme 5B).

#### 2.3 Visible-light-mediated Cu(II) catalytic cycle

**Scheme 6:**
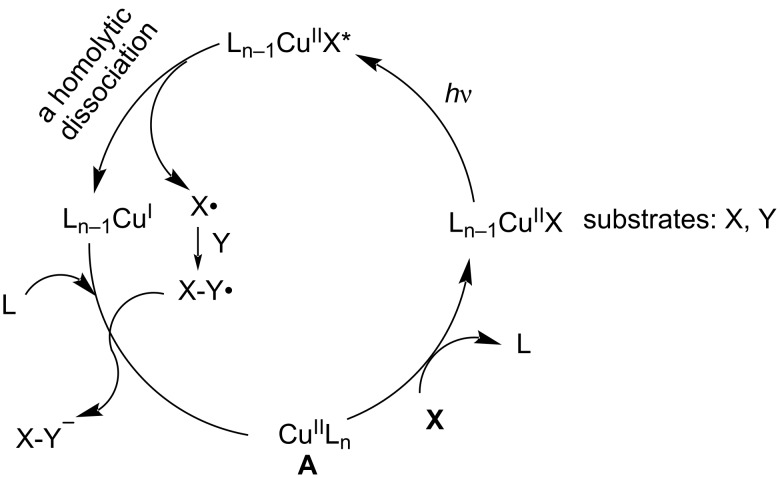
Mechanism of Cu^II^-base photocatalysis.

The precatalyst L_n_Cu^II^ (**A**) undergoes ligand exchange with substrate X to form a catalytically active species, namely, L_n−1_Cu^II^X. Upon irradiation, L_n−1_Cu^II^X is converted to a photoexcited species L_n−1_Cu^II*^X, which undergoes homolytic dissociation to produce L_n−1_Cu^I^ and radical X^•^. The radical X^•^ can add to the substrate Y to obtain the stable radical X–Y^•^. Subsequently, L_n−1_Cu^I^ transfers one electron to X–Y^•^ and accepts one ligand to regenerate the intermediate L_n_Cu^II^ and the final product [39,43] (Scheme 6).

Copper photocatalysis is a powerful tool that can be used to construct carbon–heteroatom and carbon–carbon bonds and can be applied to radical chemistry. This review discusses copper-catalyzed reactions including alkene and alkyne, organic halide, and alkyl C–H functionalization.

### 3. Visible-light-mediated copper-catalyzed alkene and alkyne functionalization

#### 3.1 Olefinic C–H functionalization and allylic alkylation

**Scheme 7:**
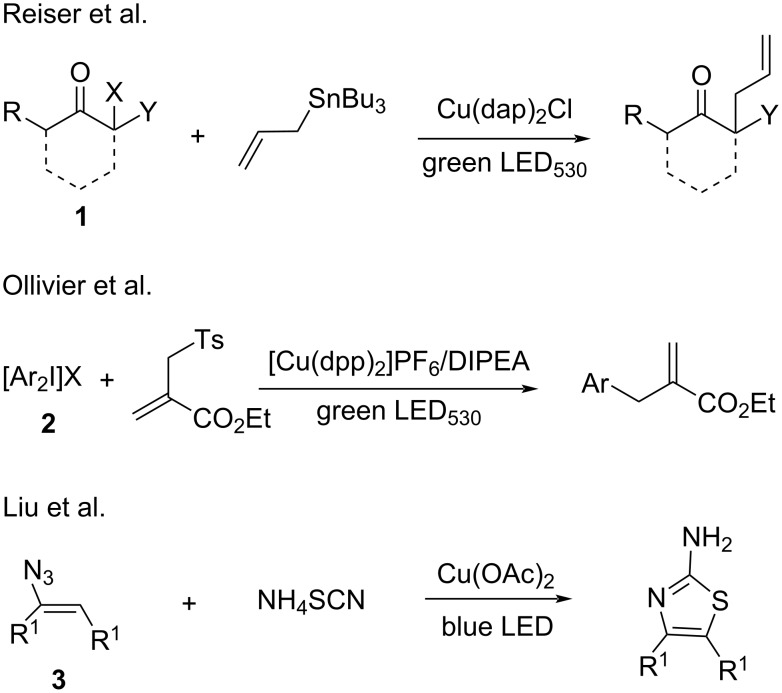
Olefinic C–H functionalization and allylic alkylation.

Under mild conditions, copper salts are able to catalyze olefinic C–H functionalization or allylic alkylation, thus allow introducing alkenyl or allyl groups into organic molecules. Alkenylation and allylation reactions have been extensively investigated under thermal conditions. However, only few studies included visible-light catalysis. In 2012, Reiser’s group [44] reported the allylation of α-haloketones **1** with olefins under irradiation (λ = 530 nm) in the presence of [Cu(dap)_2_Cl] (dap = 2,9-di(*p*-anisyl)-1,10-phenanthroline) as the catalyst. They conducted control experiments to establish that [Cu(dap)_2_Cl] and visible light are necessary for this transformation. In 2013, Ollivier and co-workers [45] successfully applied the same strategy to the allylation of diphenyliodonium **2**. In 2017, Liu’s group [46] reported the copper salt-catalyzed cyclization of vinyl azides **3** with ammonium thiocyanate to generate 4-alkyl/aryl-2-aminothiazoles. Mechanistic experiments demonstrated that the photocatalyst formed in situ from Cu(OAc)_2_ and ammonium thiocyanate promoted the intermolecular cyclization (Scheme 7).

#### 3.2 Difunctionalization of alkenes

The 1,2-difunctionalization of alkenes is a versatile strategy for the construction of complex molecules. The primary process involved in the 1,2-difunctionalization of alkenes catalyzed by copper complexes is an atom-transfer radical addition (ATRA). Copper complexes or copper-based photoredox-active complexes formed in situ serve as photocatalysts to transfer electrons to suitable radical precursors. The detailed catalytic cycle is presented in section 3.1 and involves ligand exchange and rebound mechanisms.

**Scheme 8:**
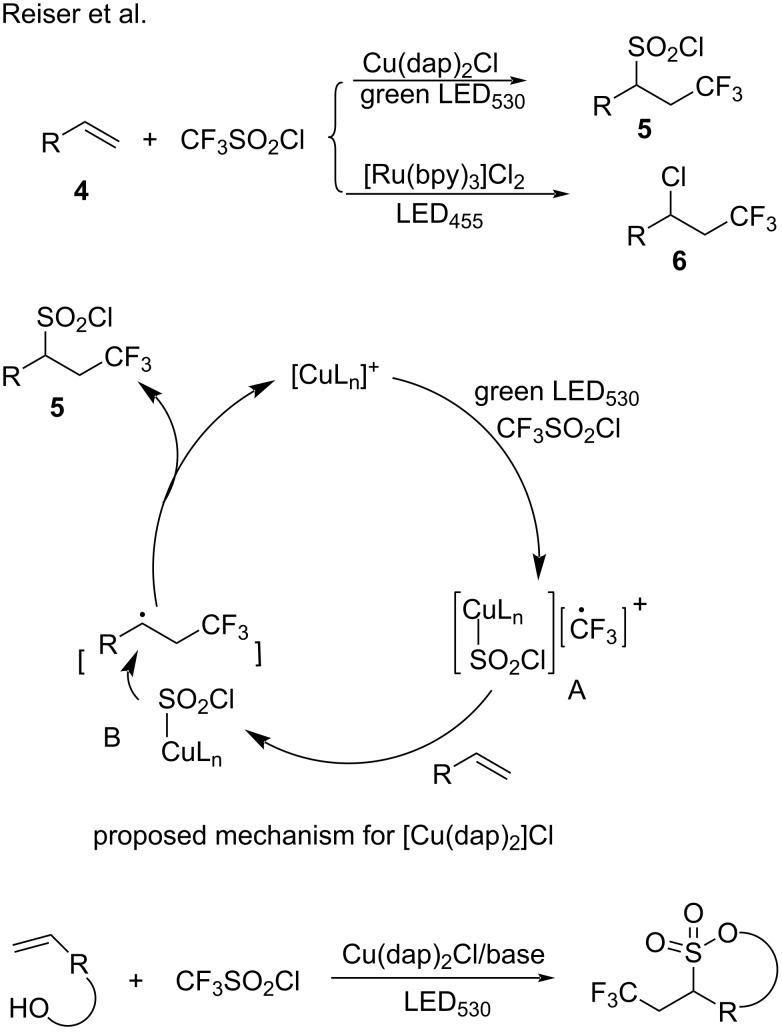
Cross-coupling of unactivated alkenes and CF_3_SO_2_Cl.

A comprehensive survey of copper photocatalysts was initiated from Reiser’s group. In 2015, Reiser and co-workers [47] reported the [Cu(dap)_2_]Cl-catalyzed trifluoromethylchlorosulfonylation of unactivated alkenes **4** under photochemical conditions. When used in place of [Cu(dap)_2_]Cl, ruthenium-based, iridium-based, and eosin Y catalysts promoted the trifluoromethylchlorination of alkenes with the extrusion of SO_2_ (e.g., product **6**). Studies were performed to elucidate the strikingly different reactions that occurred with these different photoredox catalysts. The catalytic cycle of [Cu(dap)_2_]Cl is shown in Scheme 8. In this catalytic cycle, [Cu(dap)_2_]Cl excited by irradiation with visible light reacts with triflyl chloride in a SET process to generate a trifluoromethyl radical and L_n_Cu^II^SO_2_Cl (intermediate A in Scheme 8). The formed trifluoromethyl radical adds to the alkene moiety to deliver a new alkyl radical, which is trapped by the L_n_Cu^II^-SO_2_Cl species. Free SO_2_Cl**^–^** decomposes rapidly to SO_2_ and Cl**^–^**. However, in this transformation, SO_2_Cl**^–^** is stabilized by the copper complex. The alkyl radical reacts with L_n_Cu^II^-SO_2_Cl to deliver the target product **5**. A mechanistic study demonstrated that [Cu(dap)_2_]Cl can coordinate with the reactive intermediate SO_2_Cl and suppresses the extrusion of SO_2_. Thus, [Cu(dap)_2_]Cl achieves a unique transformation under visible-light irradiation by acting as a catalyst for single-electron reduction and as an intermediate stabilizing agent (Scheme 8). In 2016, the same group [48] reported the [Cu(dap)_2_]Cl-catalyzed cyclization of α,ω-alkenols and trifluoromethylsulfonyl chloride to form sultones (Scheme 8).

**Scheme 9:**
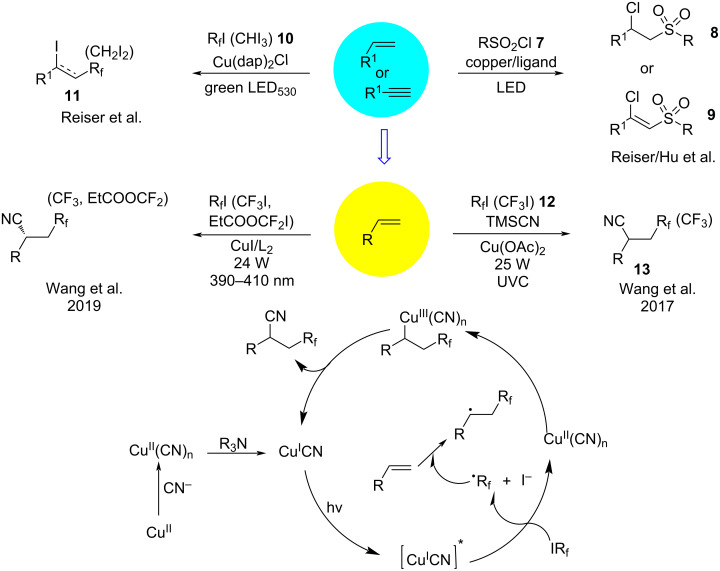
Chlorosulfonylation/cyanofluoroalkylation of alkenes.

Intrigued by this unique transformation, Reiser’s group [49] extended this protocol to the chlorosulfonylation of alkenes and alkynes in 2019. Under visible light irradiation and in the presence of [Cu(dap)_2_]Cl, the reaction of *p*-toluenesulfonyl chloride (**7**) with alkenes gave an excellent yield of the chlorosulfonylated products **8** and **9**, whereas replacing the copper catalyst by ruthenium-based, iridium-based, and eosin Y catalysts afforded the desired products only in trace amount. Unexpectedly, the corresponding Cu^II^ complex, Cu(dap)Cl_2_, also produced the desired product with good yield. Based on the literature [28,50], Cu(dap)Cl_2_ acted as a potential precatalyst in this photoreaction. Upon irradiation, the Cu^II^ complex undergoes homolytic cleavage of a Cu–Cl bond forming Cu^I^ as the catalytically active species; thus, the Cu^II^ complex is the precatalyst and provides a more efficient transformation than Cu^I^. Under optimized conditions, the substrate scope was examined and determined to include activated olefins, unactivated olefins, and arylalkynes. In parallel, Hu and co-workers [51] reported the photoinduced, copper-catalyzed chlorosulfonylation of alkenes and alkynes under irradiation with blue LEDs. Reiser and co-workers [52–53] unexpectedly observed the iodoperfluoroalkylation of alkenes and perfluoroalkyl iodides **10** in the presence of [Cu(dap)_2_]Cl. Consistent with the previous report, the desired products **11** were not obtained with ruthenium-based, iridium-based, and eosin Y catalysts (Scheme 9), which was due to the ability of copper to stabilize and interact with radical intermediates in its coordination sphere. Mechanistic studies revealed that the iodoperfluoroalkylation of alkenes and alkynes involved a rebound or ligand transfer cycle (section 3.1). In 2017, Wang and co-workers [54] discovered the photoinduced, copper-catalyzed cyanofluoroalkylation of alkenes and fluoroalkyl iodides **12**. The reaction was initiated by the reduction of Cu^II^ with tertiary amines, which formed Cu^I^CN and an amine radical cation [55]. Under irradiation by ultraviolet light, Cu^I^CN was excited and transformed to its triplet state Cu^I^CN^*^, in which the fluoroalkyl iodides were reduced to R_f_^•^ and I^−^. Subsequently, the radical R_f_^•^ attacks the alkene forming a new alkyl radical species. This radical species is then trapped by Cu^II^(CN)_n_ to generate a Cu^III^ intermediate, which undergoes reductive elimination to form the desired product **13** (Scheme 9). In 2019, the same group [56] applied this protocol to the asymmetric cyanofluoroalkylation of alkenes. Under visible-light irradiation, the Cu-based catalyst plays a dual role as both the photosensitizer for the SET and the catalyst for asymmetric control (Scheme 9).

**Scheme 10:**
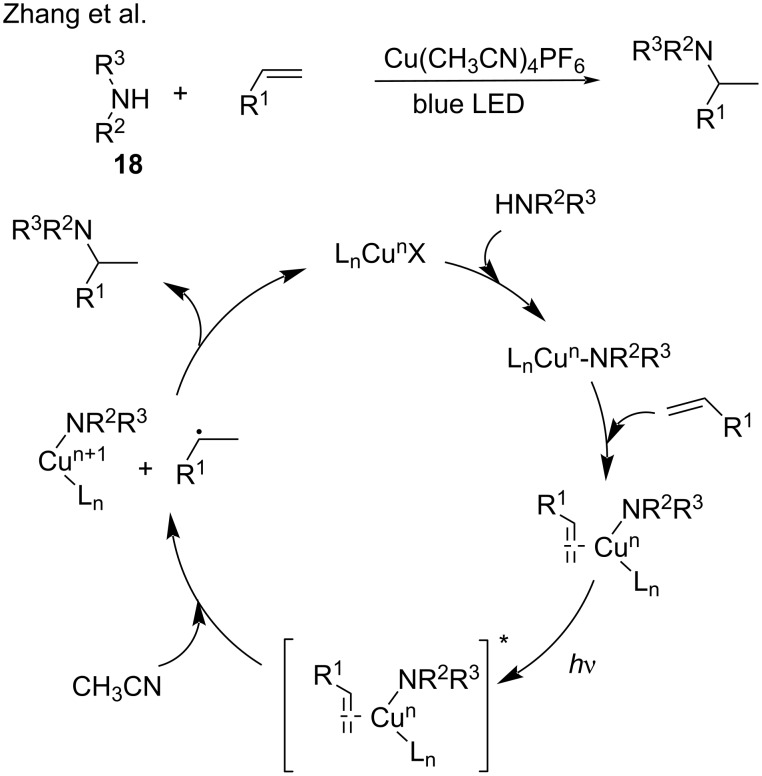
Hydroamination of alkenes.

**Scheme 11:**
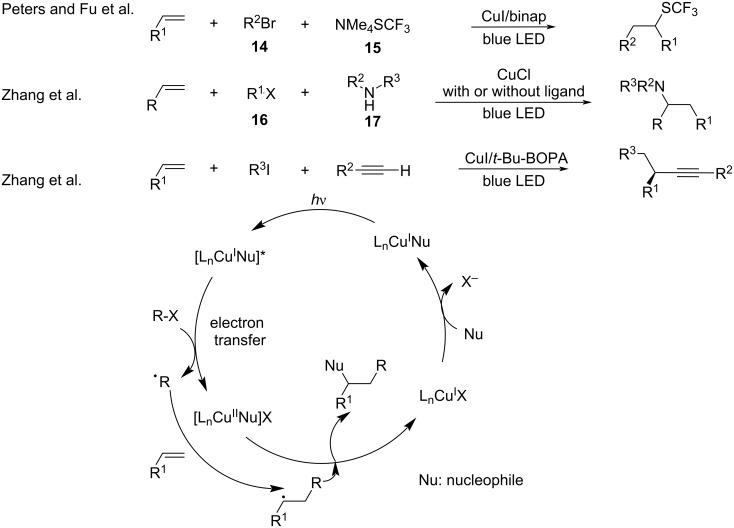
Cross-coupling reaction of alkenes, alkyl halides with nucleophiles.

In addition to perfluoroalkyl iodides, this protocol was further extended to alkyl halides, trifluoromethylthiolate, amines, cycloketone oxime esters, and carboxylic acid *N*-hydroxyphthalimide esters (NHPI). In 2018, Peters and Fu [57] explored the copper-catalyzed three-component coupling of alkyl halides **14**, olefins, and trifluoromethylthiolate **15**. Mechanistic studies demonstrated that the photoexcited Cu^I^/binap/SCF_3_ complex generated in situ engages in electron transfer with the alkyl halides, thereby providing an alkyl radical and the Cu^II^/binap/SCF_3_ species. Subsequently, the alkyl radical reacts with the olefin generating a new alkyl radical, which is trapped by Cu^II^/binap/SCF_3_ to provide the coupling product (Scheme 10). In 2019, Zhang and co-workers [58] reported the photoinduced copper-catalyzed carboamination of alkenes that involved organic halides **16**, alkenes, and amines **17**, **18** (Scheme 10 and Scheme 11). Based on previous mechanistic studies [41], the authors found that the photoexcited ligand–Cu^I^−amido species transferred electrons to alkyl halides to produce alkyl radicals, which reacted with alkenes and amines to generate the three-component coupling products. In the absence of organic halide, the copper salts catalyzed the hydroamination of the alkene [59]. Mechanistic studies showed that the copper–amido complex coordinated with alkenes, which then acted as a primary photocatalyst. After light irradiation, the excited alkene–copper–amido species offered a benzyl radical and the organocopper via SET with hydrogen atom abstraction from CH_3_CN. Subsequently, the benzyl radical was captured by the organocopper to generate the hydroamination products (Scheme 10). In 2020, the same group [60] reported the copper-catalyzed asymmetric dual carbofunctionalization of alkenes with alkynes and alkyl halides (Scheme 11). The alkynyl copper-ligand served as the photoactive species and delivered a single electron to the alkyl halide to produce the alkyl radical, which then reacted with the alkene and alkyne to generate the coupling products (Scheme 10).

**Scheme 12:**
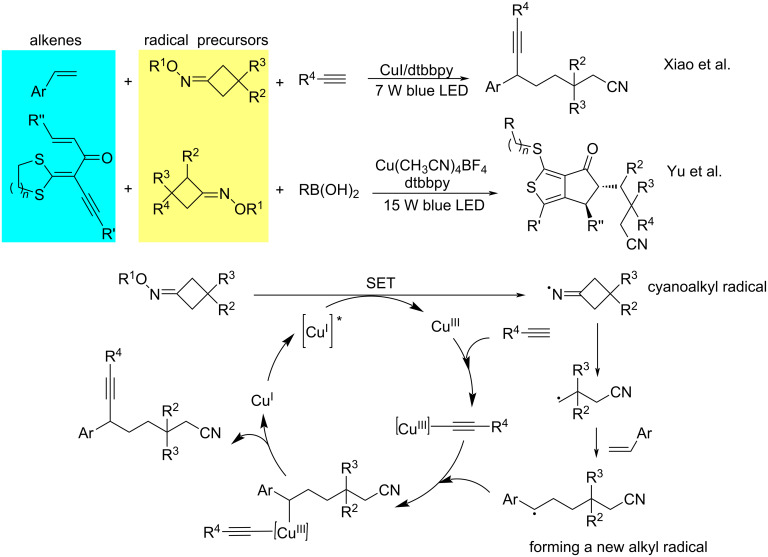
Cross-coupling of alkenes with oxime esters.

From 2018 to 2020, Xiao and Yu et al. [61–62] disclosed a series of copper-catalyzed cyanoalkylation reactions among alkenes, oxime esters, and boronic acids or alkynes. Mechanistic studies implied that the Cu^I^ complex gets photoexcited via a SET process to generate a cyanoalkyl radical from the oxime esters. The resulting cyanoalkyl radical then adds to the alkene to form a new alkyl radical. This radical is captured by a high-valent Cu^III^ complex, which undergoes a reductive elimination to give the target product (Scheme 12).

**Scheme 13:**
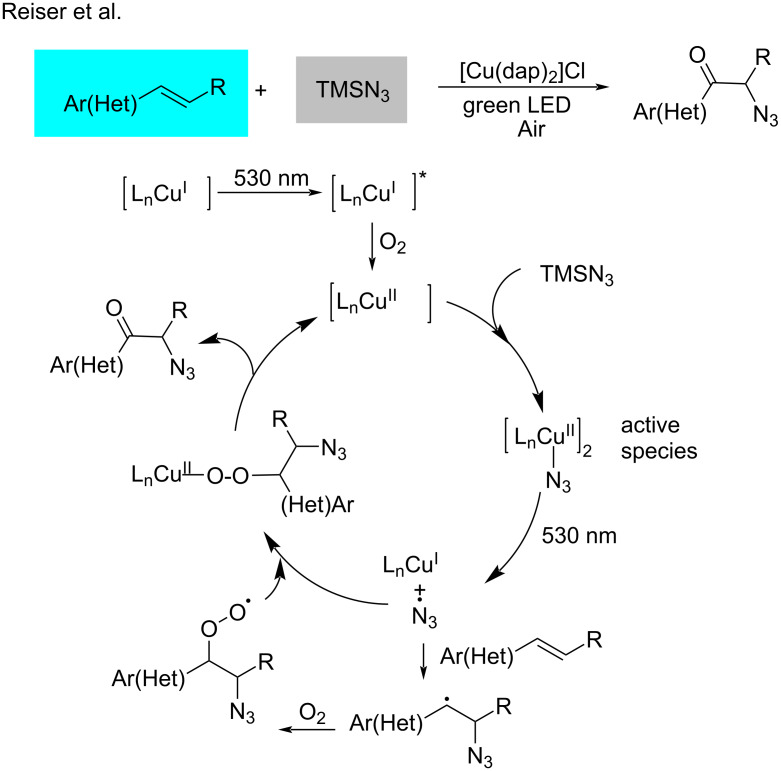
Oxo-azidation of vinyl arenes.

**Scheme 14:**
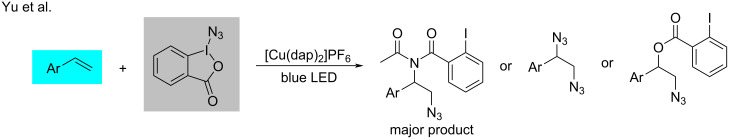
Azidation/difunctionalization of vinyl arenes.

In 2018, Reiser and co-worker [63] established a Cu^II^-catalyzed oxo-azidation of vinyl arenes (Scheme 13). In this transformation, the azide anion is oxidized to its radical, and this process requires a high reduction potential that cannot be achieved by iridium and ruthenium-based catalysts. In contrast, [Cu(dap)_2_]Cl and [Cu(dap)Cl_2_] were found suitable for the oxidation. Based on a mechanistic study, Cu^II^ serves as the catalytically active species that undergoes homolytic cleavage to form a Cu^I^ species and an azide radical. The latter adds to the alkene to form an alkyl radical, which is then trapped by oxygen to form the desired product. The homolytic cleavage of the active species represents a new platform for copper-based photocatalysis (see section 3.3). In 2019, Yu et al. [64] developed a similar copper-catalyzed azidation of activated alkenes with 1-azido-1,2-benziodoxole as the azide radical precursor (Scheme 14).

#### 3.3 Functionalization of alkynes

**Scheme 15:**
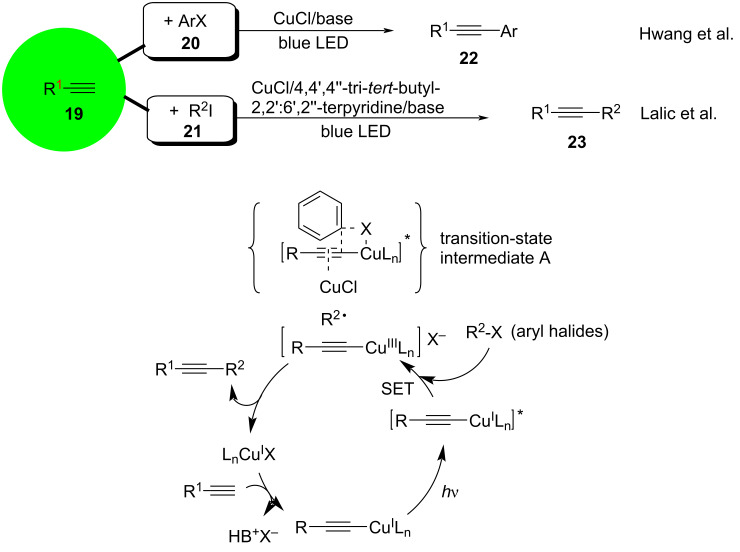
Photoinitiated copper-catalyzed Sonogashira reaction.

A series of photoinduced copper-catalyzed coupling reactions of terminal alkynes was published by Hwang and co-worker. In 2012, they [65] achieved a photoinitiated Sonogashira reaction using aryl halides (bromides and iodides) **20** and aryl- or alkylacetylenes **19**. In control experiments, the replacement of the copper salt with palladium salts or the substitution of thermal heating for LED irradiation resulted in a low yield of the cross-coupling products **22** and **23**, indicating the necessity of the copper salt and LED irradiation. Mild conditions, such as Pd-free and high reaction yields at room temperature, make this method a very promising, scalable green process that can be used as an alternative to the conventional Sonogashira cross-coupling reactions. In 2018, Lalic and co-workers [66] extended this approach to alkyl halides and reported the photoinduced copper-catalyzed Sonogashira coupling of alkynes and alkyl iodide **21**. The proposed mechanism is shown in Scheme 15. Under blue visible light, the haloalkane is reduced by the copper acetylide to form the alkyl radical intermediate R_2_^•^. If aryl halide is the haloalkane, the copper acetylide is attacked by the aryl halide to form transition-state intermediate A. The copper acetylide is transformed into a high-valent Cu^III^ complex, which subsequently undergoes reductive elimination or dissociation of the transition-state intermediate in the case of aryl halide to generate the target product (Scheme 15).

**Scheme 16:**
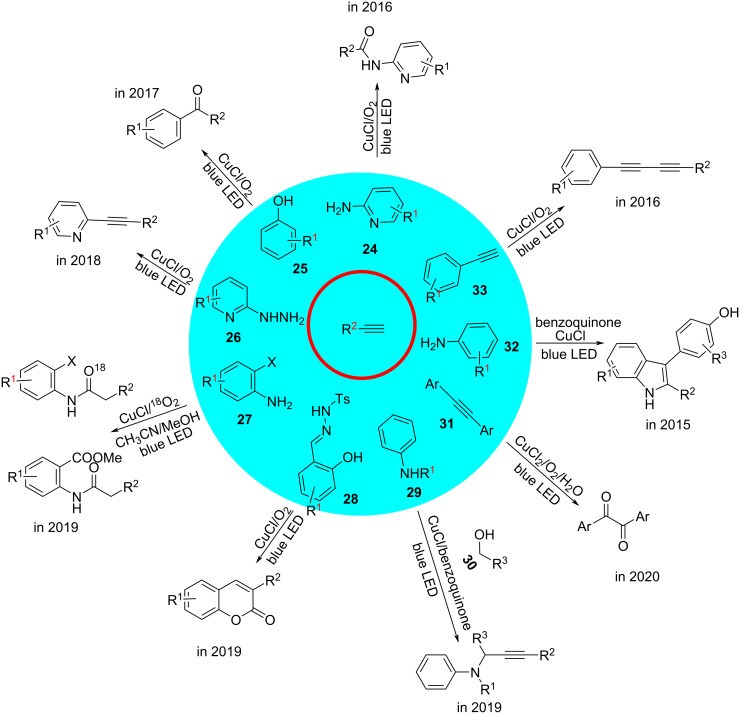
Alkyne functionalization reactions.

From 2015 to 2020, Hwang and co-workers [67–71] investigated the copper-catalyzed oxidative coupling of alkynes, nucleophiles (e.g., phenols **25** and **28**; amines **24**, **27**, **29**, and **32**; 2-hydrazinylpyridine **26**; alkyne **33**; and alcohol **30**), and oxidants (benzoquinone or O_2_). Based on the literature and mechanistic experiments [72–73], the reaction is initiated by the photoirradiation of in situ-generated Cu^I^ phenylacetylide. From the excited state of Cu^I^ phenylacetylide an electron is transferred to the oxidants (benzoquinone or O_2_) via a SET, thereby forming a Cu^II^ phenylacetylide species and a radical anion. The resulting Cu^II^ phenylacetylide species is involved in the bond-forming reaction [74]. As a notable exception, in 2016, Hwang’s group [75] reported the novel synthesis of unsymmetrical 1,3-conjugated diynes **31** from terminal alkynes under LED irradiation. The reaction mechanism involved a bipolar heterodimeric copper phenylacetylide species that showed similar photophysical properties (Scheme 16).

**Scheme 17:**
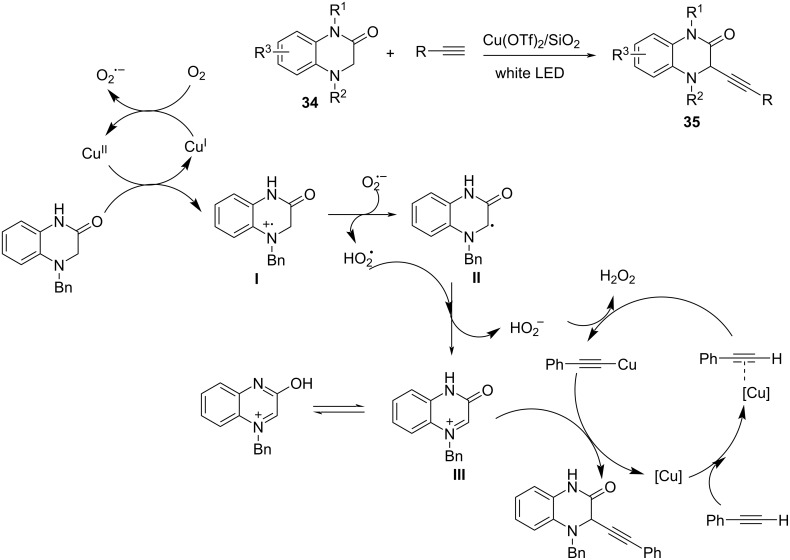
Alkynylation of dihydroquinoxalin-2-ones with terminal alkynes.

In 2019, Vlla’s group [76] explored the copper-catalyzed alkynylation of dihydroquinoxalin-2-ones **34** with terminal alkynes under irradiation. 4-Benzyl-3,4-dihydroquinoxalin-2(1*H*)-one **35** was subjected to an oxidation process with a Cu^II^ salt to generate a nitrogen radical cation **I** and a Cu^I^ species. This process regenerated Cu^II^ in the presence of molecular oxygen. The deprotonation of the nitrogen radical cation produces an α–amino radical **II**, which was further oxidized to the iminium ion **III** to which the copper alkynylide added forming the desired product (Scheme 17).

**Scheme 18:**
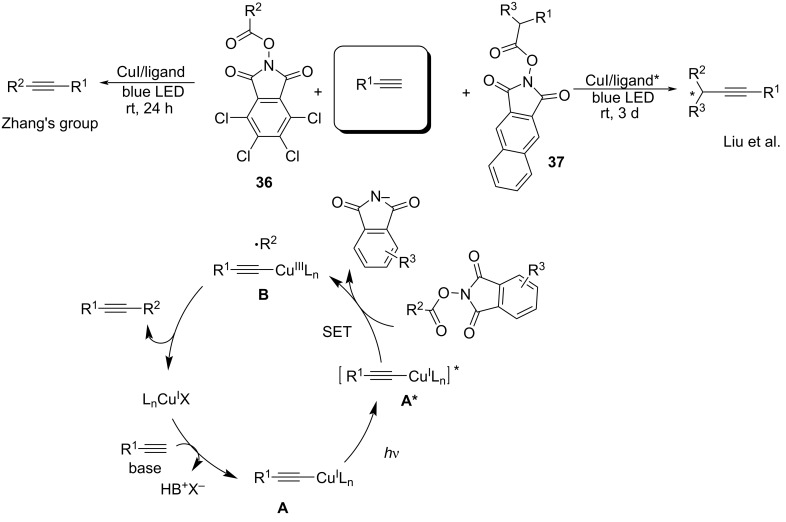
Decarboxylative alkynylation of redox-active esters.

In 2020, Zhang’s group [77] described the photoinduced copper-catalyzed decarboxylative alkynylation of redox-active esters with terminal alkynes. *N*-Hydroxy-tetrachlorophthalimide (TCNHPI, **36**) derived from carboxylic acids was identified as the ideal radical precursor. Under irradiation, the Cu^I^ acetylide–ligand species **A** generated in situ was irradiated to form the activated state A^*^, which transferred a single electron to TCNHPI to form an alkyl radical and tetrachlorophthalimide anion and concurrently generated the oxidative Cu^I^ acetylide species **B**. The intermediate **B** was subsequently trapped by the alkyl radical and underwent reductive elimination to deliver the desired product. Liu’s group [78] further applied this protocol to the asymmetric decarboxylative alkynylation of *N*-hydroxy 2,3-naphthalimide-derived ester **37** with terminal alkynes. Remarkably, the *N*-hydroxy 2,3-naphthalimide-derived ester acted as an ideal radical precursor and accepted a single electron from the excited state Cu^I^-acetylide complex. The copper catalyst plays a dual role, namely, as a photoredox catalyst and a cross-coupling catalyst. NHP-type esters inhibited the homodimerization of the alkyl radical and terminal alkyne (Scheme 18).

**Scheme 19:**
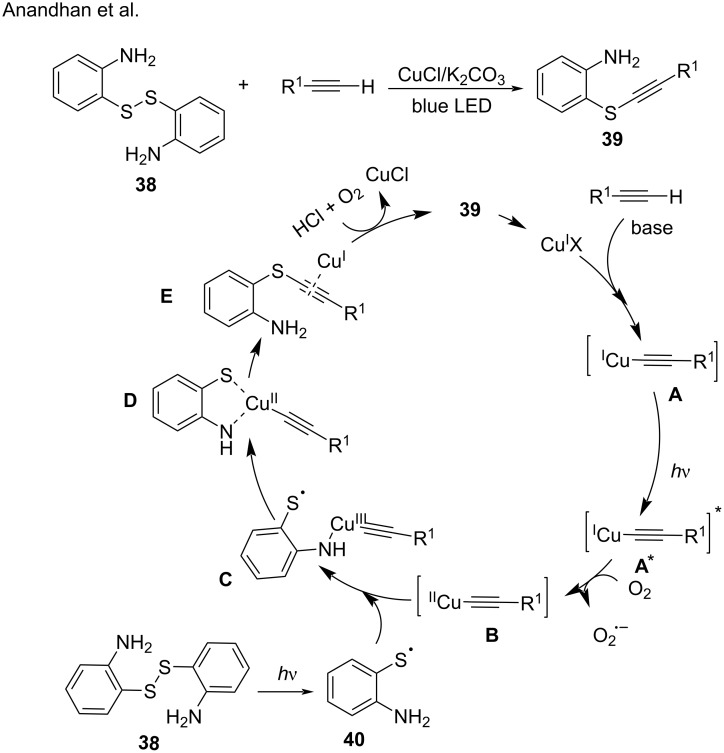
Aerobic oxidative C(sp)–S coupling reaction.

Under visible-light irradiation, disulfides are easy transformed to thiyl radicals via the homolytic cleavage of the S–S bond [79]. In 2020, Anandhan and co-workers [80] explored the C(sp)–S coupling of terminal alkynes with 2-aminothiophenol dimer **38** as a radical precursor. Under photoexcitation the Cu^I^ acetylide **A** undergoes a SET process to form the Cu^II^ phenylacetylide species **B** and a superoxide radical anion. In parallel, under irradiation the homolytic S–S-bond cleavage in 2-aminothiophenol dimer **38** forms thiol radicals **40**. The nucleophilic addition of the amino group in radical **40** to the Cu^II^ acetylide **B** generates the Cu^III^ acetylide species **C**, which coordinates with the thiol radical to give the Cu^II^-aminothiophenol complex **D**. Finally, the intermediate Cu^I^-aminothiophenol complex **E** reacted with HCl and O_2_ to generate the C(sp)-S coupling product **39** (Scheme 19).

#### 3.4 Functionalization of organic halides

As demonstrated by the different photoredox mechanisms of Cu complexes, the Cu^I^ complexes have strong reduction ability and can promote electron transfer to organic substrates. Thus, the high reduction potentials of Cu^I^ complexes are also applied to the functionalization of organic halides. The seminal work by Fu and Peters demonstrated that with the help of light, copper–nucleophile complexes undergo excitation, and the resulting complex engages with organic halides in a SET process to generate alkyl or aryl radicals. Next, a C–X (X = O, N, S, C) bond is formed between the nucleophile and the alkyl or aryl radicals. From 2013 to 2019, the authors disclosed a series of nitrogen, sulfur, oxygen, and carbon nucleophiles for photoinduced, copper-catalyzed cross-couplings with organic halides. The copper–nucleophile complexes that were generated in situ as photoredox catalysts transferred electrons to organic halides, thereby achieving the cross-coupling. The detailed mechanistic studies are shown in section 3.2.

**Scheme 20:**
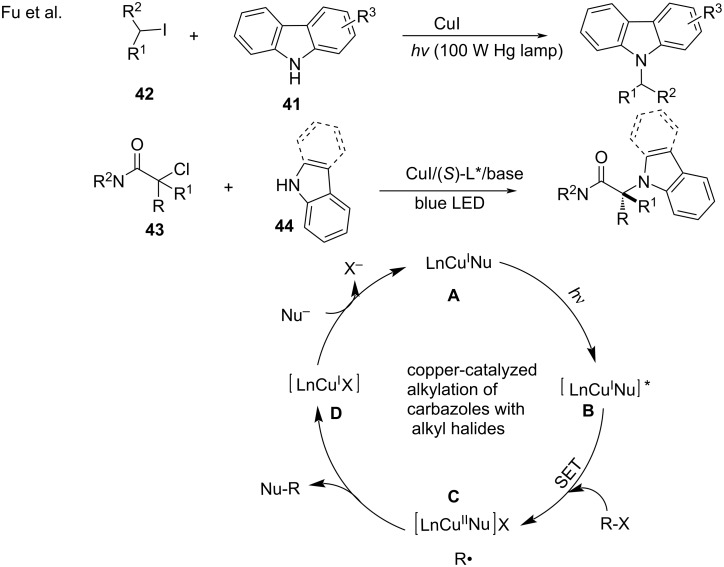
Copper-catalyzed alkylation of carbazoles with alkyl halides.

In 2013, Fu and co-workers [81] reported the photoinduced copper-catalyzed alkylation of carbazoles **41** with alkyl halides **42** and completed the corresponding mechanistic study [41–42] in 2017. This metal-catalyzed, photoinduced, and asymmetric radical transformation requires two catalysts, namely, (i) a metal catalyst that promotes electron transfer and (ii) a separate chiral catalyst that facilitates the highly stereoselective bond formation. In 2016, Fu [82] discovered the asymmetric cross-coupling of racemic tertiary alkyl halides **43** with carbazoles or indoles **44** in the Cu^I^/chiral phosphine system. Under irradiation conditions, excitation of the copper–nucleophile complex **A** results in the excited state species **B** that engages in the electron transfer with the alkyl halide to generate a copper^II^–nucleophile complex **C** and an alkyl radical. The formation of the R–Nu bond might occur through an in-cage pathway involving complex **C** (Scheme 20).

**Scheme 21:**
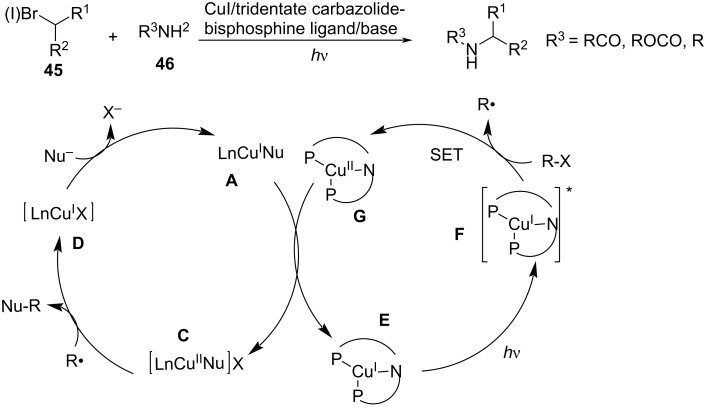
C–N coupling of organic halides with amides and aliphatic amines.

In addition to carbazoles, the authors further described the C–N coupling of organic halides **45** with amides [83] and aliphatic amines [84] **46**. The results of the mechanistic studies showed that a copper/tridentate carbazolide-bisphosphine ligand complex serves as a new photoredox catalyst engaged in the electron transfer to the electrophile. Under photoexcitation, the excited photoredox catalyst **F** reduces the alkyl halide, producing an alkyl radical and a copper^II^ intermediate **G**, which oxidizes a copper^I^–nucleophile complex **A** to the corresponding copper^II^–nucleophile complex **C**. Complex **C** then couples with the alkyl radical to generate the product Nu–R in an out-of-cage process (Scheme 21).

**Scheme 22:**
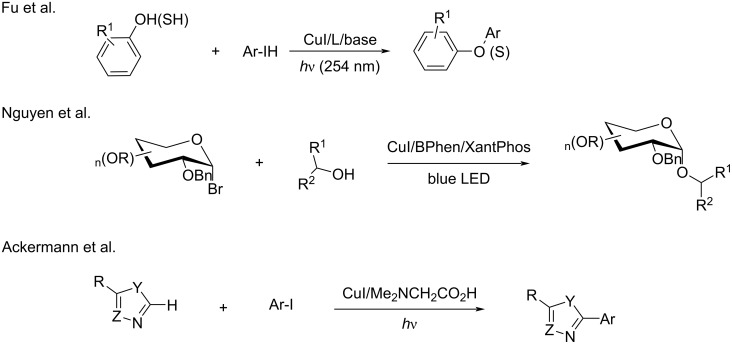
Copper-catalyzed C–X (N, S, O) bond formation reactions.

The same group was interested in extending this protocol from C–N bond formation reactions to C–O [85], C–S [86], and C–C [87–88] bond formations. Recently, a photoredox catalysis was applied to these types of cross-coupling reactions, with key contributions from the groups of Ackermann [87], Evano [55], Zhang [89], Nguyen [90], and Bissember [91]. In 2013, Peters’ group [86] established the copper-catalyzed C–S cross-coupling between thiols and aryl halides. The mechanistic studies revealed that the reaction runs with the inexpensive precatalyst (CuI) and no ligand co-additive is necessary. In 2014, the same group [85] reported the copper-catalyzed C–O cross-coupling reaction. Results of mechanistic studies indicated that in this case a Cu^I^–phenoxide complex is a competent intermediate in the photoinduced C–O bond formation. In 2020, Nguyen and co-worker [90] reported copper-catalyzed C–O cross-coupling of glycosyl bromides with aliphatic alcohols. In 2015, Ackermann’s group [88] disclosed the visible light-induced copper-catalyzed arylation of azoles. In this case, the mechanistic studies revealed that amino acid ligands accelerated the cross-coupling (Scheme 22).

**Scheme 23:**
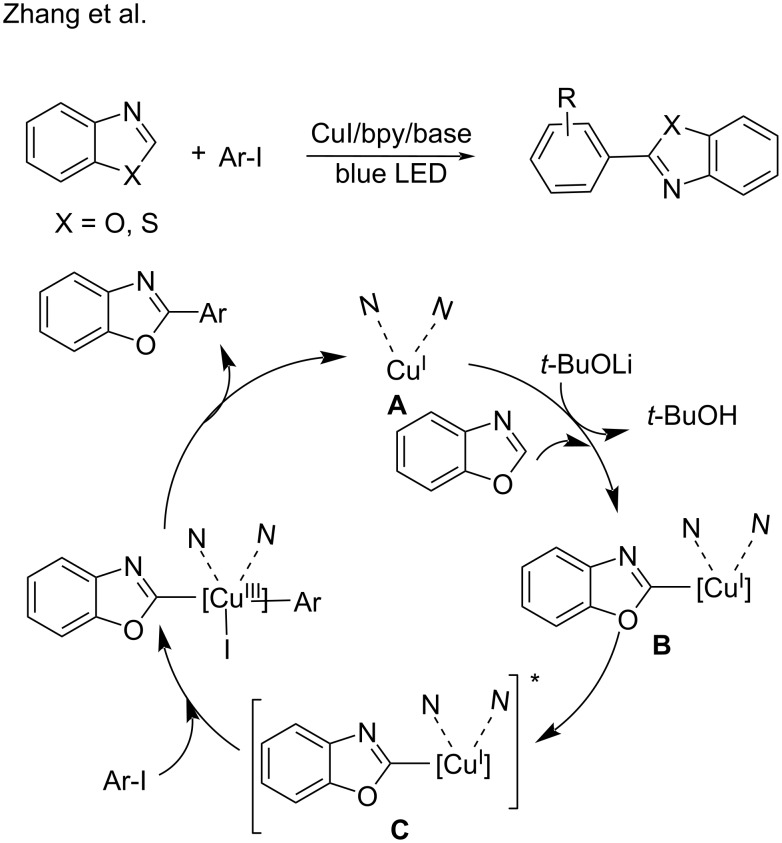
Arylation of C(sp^2^)–H bonds of azoles.

In 2020, a visible-light-induced copper-catalyzed arylation of C(sp^2^)–H bonds of azoles was developed by Zhang [89]. A 2,2’-bipyridine copper coordination compound served as the photoredox catalyst and accomplished the azole C–H arylations. Under irradiation with blue LED, the photoexcited state [L_n_Cu^I^-benzoxazole]^*^ (**C**) engages in a double electron-transfer process with aryl iodides to generate intermediate **D**, which then undergoes reductive elimination to generate the desired products (Scheme 23).

**Scheme 24:**
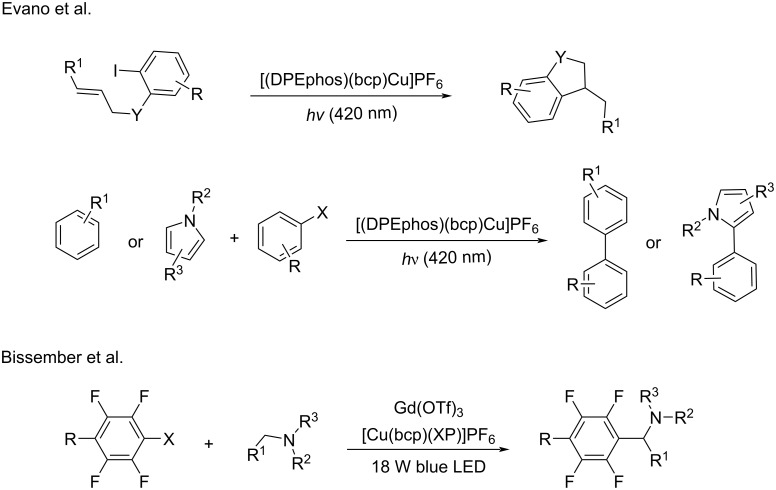
C–C cross-coupling of aryl halides and heteroarenes.

In 2017, Evano’s group [55] established a photoinduced, copper-catalyzed C–C cross-coupling of aryl halides, and heteroarenes. The cyclization of *N*-allyl-*o*-iodoanilines was further studied in intramolecular processes. [(DPEphos)(bcp)Cu]PF_6_, as a photocatalyst, was applied in these transformations. In 2018, the Bissember group [91] reported the photoinduced and copper-catalyzed dual α-amino-C–H/C–F functionalization reaction (Scheme 24).

#### 3.5 Alkyl C–H functionalization reactions

**Scheme 25:**
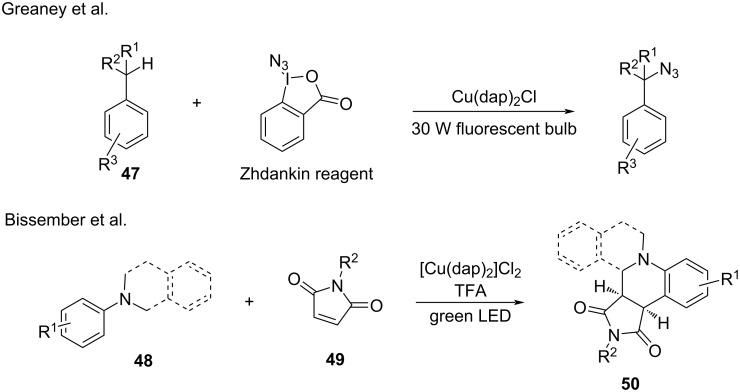
Benzylic or α-amino C–H functionalization.

Benzylic or α-amino C–H groups and even the stable C(sp^3^)–H group were functionalized through the corresponding benzylic radical, α-amino radical, or alkyl radical. In 2016, Greaney and co-workers [92] investigated the direct C–H azidation with benzylic C–H compounds **47** and the Zhdankin reagent. After investigating a range of reaction parameters, copper salts and visible light were found to be necessary for the transformation. The reaction is highly selective for the benzylic position. In the same year, Bissember’s group [93] reported a copper-photocatalyzed α-amino C–H functionalization. In this work, *N*,*N*-dialkylanilines or *N*-aryltetrahydroisoquinolines **48** reacted with N-substituted maleimide **49** via annulation to provide a range of tetrahydroquinolines or tetrahydroisoquinolines **50**, respectively, with good yield. The mechanistic investigation revealed that an α-amino radical undergoes radical addition with the N-substituted maleimide (Scheme 25).

**Scheme 26:**
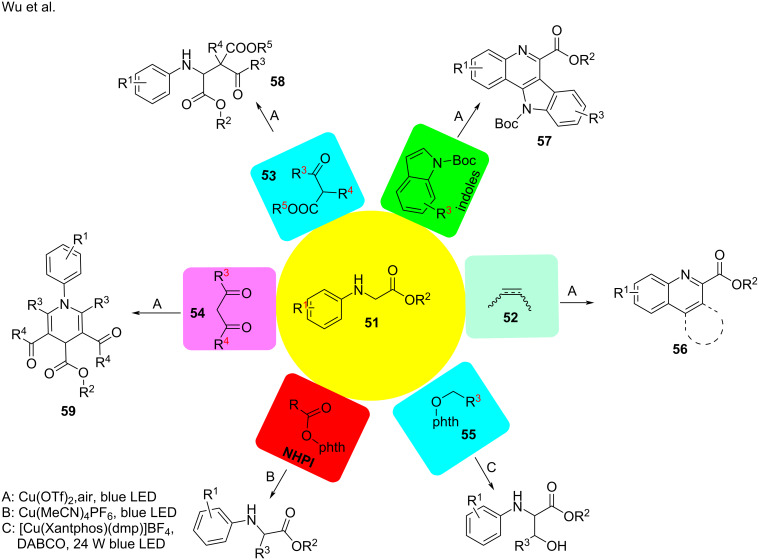
α-Amino C–H functionalization of aromatic amines.

**Scheme 27:**
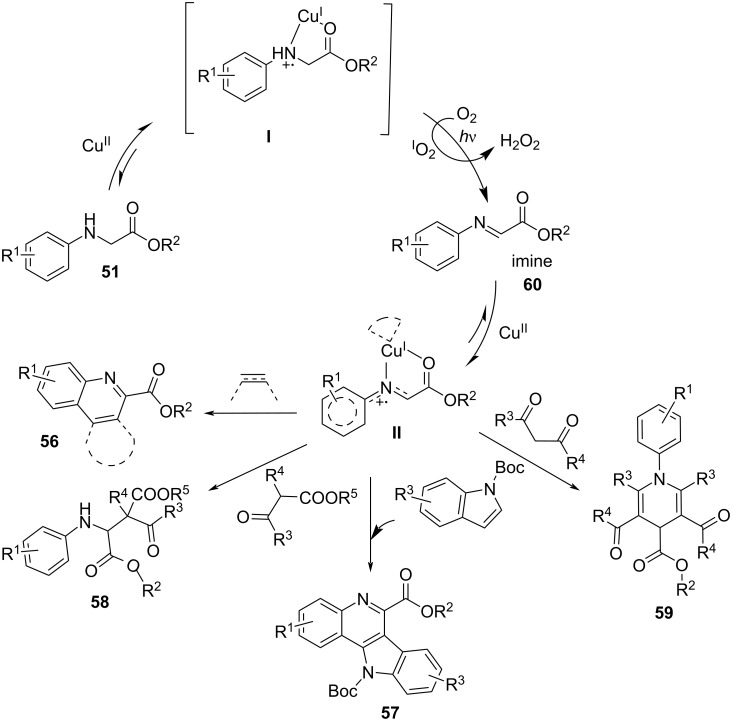
C–H functionalization of aromatic amines.

In 2017, Wu and co-workers [94] reported the α-amino C−H functionalization of aromatic amines **51** with nucleophiles, including arynes or aromatic olefins **52**, indoles, acyclic β-ketoester **53**, and β-diketone **54** (Scheme 26). Mechanistic observations revealed a new avenue for copper-based photocatalysis. The transformation was initiated by a SET process from the amine to the Cu^II^ ion to generate the visible-light-driven species **I** [Cu^I^-NH^•+^]. Under visible-light irradiation, the intermediate [Cu^I^-NH^•+^] was oxidized to the imine **60** by O_2_. Next, the imine **60** transferred a single electron to the Cu^II^ ion, thereby providing intermediate **II** [Cu^I^-N^•+^]. [Cu^I^-N^+•^] was equal to Cu^I^ and N^+•^ of the imine, which is activated for the nucleophilic addition. With the help of O_2_, Cu^I^ regenerates Cu^II^ to complete the catalytic cycle (Scheme 27). A series of quinolones **56**, indolo[3,2-*c*]quinolines **57**, β-amino acids **58**, and 1,4-dihydropyridine derivatives **59** were obtained through this route in moderate yields (Scheme 26). Besides nucleophiles, aromatic amines also reacted with redox-active radical precursors, such as NHPI [95] and *N*-alkoxyphthalimides **55** [96].

**Scheme 28:**
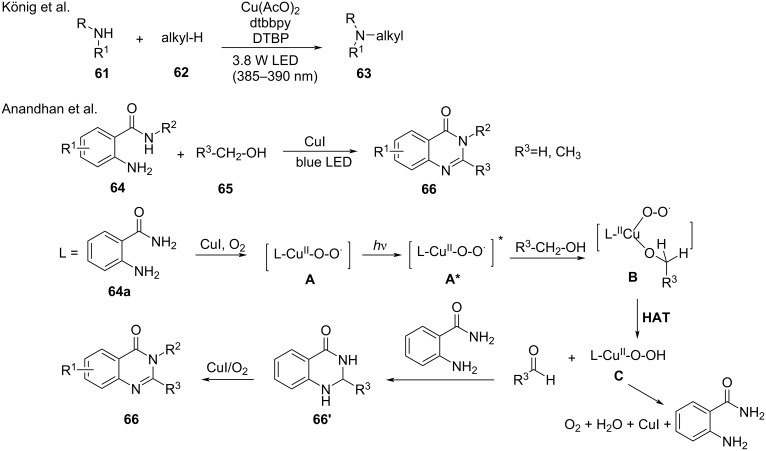
α-Amino-C–H and alkyl C–H functionalization reactions.

The functionalization of stable alkanes C(sp3)–H is generally difficult. In 2020, the König group [97] explored the photoinduced copper^II^ catalyzed N–H alkylation of a broad range of nitrogen-containing compounds **61** with unactivated alkanes **62**. A *tert*-butoxy radical abstracted a hydrogen atom from the alkane via the photolysis of DTBP producing an alkyl radical, which reacted with nitrogen-containing compounds to give the target products **63**. The catalytic cycle involves a photoinduced copper^II^ peroxide system with an in situ-generated Cu^II^–N complex as the key catalytic species. In 2020, Anandhan’s group [98] developed photoinduced copper-catalyzed α-C(sp^3^)–H cyclization of aliphatic alcohols with *o*-aminobenzamide. However, the aliphatic alcohols were limited to methanol and ethanol. In this transformation, α-C(sp^3^)–H of MeOH/EtOH undergoes a hydrogen atom transfer (HAT) process to synthesize quinazolinones involving ligand-Cu^II^ superoxo complexes **A**. Under light irradiation, complex **A** produces the excited-state ligand-Cu^II^ superoxo complex A*, which undergoes coordination with the aliphatic alcohol to form complex **B**. The latter initiates the oxidation reaction and transfers a hydrogen atom from α-C(sp^3^)–H of the alcohol to generate the Cu^II^ hydroperoxo complex **C** and the corresponding aldehyde. Complex **C** can undergo a reductive elimination to recover **64a**. The liberated aminobenzamide **64a** and the aldehyde undergo a condensation reaction to produce quinazolinone **66**′, followed by oxidation with molecular oxygen to produce the desired quinazolinone **66** (Scheme 28).

#### 3.6 Other copper-photocatalyzed reactions

**Scheme 29:**
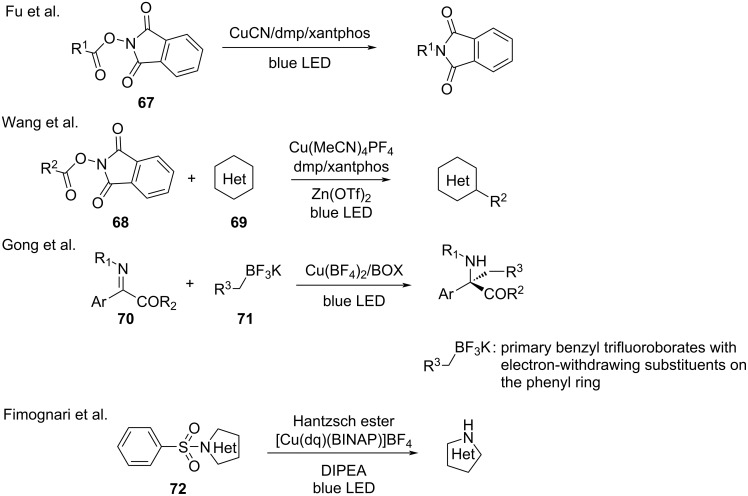
Other copper-photocatalyzed reactions.

With the advances in photocatalyzed reactions, radical precursors have received considerable research attention as practical and mild functional reagents. Extensive studies have been reported. The Fu [99] and Wang [100] groups reported that NHPI esters (**67**, **68)** have been used as alkyl radical precursors in decarboxylative coupling reactions. These reactions feature a wide substrate scope. Primary, secondary, and tertiary alkyl carboxylic acids exhibit good yield, such as decarboxylative coupling reactions between N-heteroarenes **69** and redox-active esters **68**. In 2018, Gong and co-workers [43] used benzyltrifluoroborates **71** as a benzylic radical source for the visible-light-induced alkylation of imines **70**. In the catalytic system, chiral ligands initiated benzylic radical formation and governed the subsequent stereoselective transformations. In addition, Fimognari’s group [101] utilized copper photoredox catalysts to achieve the N-desulfonylation of benzenesulfonyl-protected N-heterocycles **72** (Scheme 29).

**Scheme 30:**
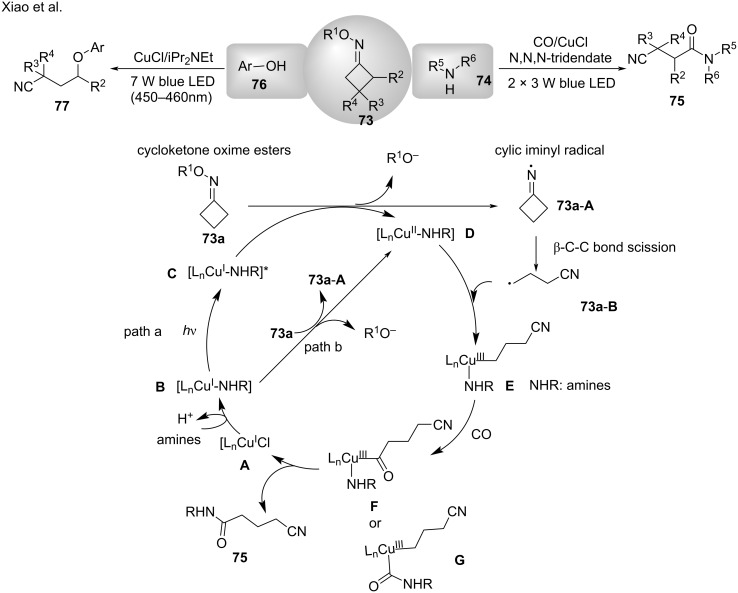
Cross-coupling of oxime esters with phenols or amines.

In 2019, Xiao’s group [102] observed that under visible light or copper catalysis, cycloketone oxime esters **73** formed cyclic iminyl radicals, which then formed cyanoalkyl radicals through a selective β-C–C bond scission. This protocol was further applied to the aminocarbonylation of cycloketone oxime esters with CO gas and amines **74**. Cycloketone oxime esters are reduced by the photoexcited [L_n_Cu^I^–NHR]^*^ complex **C** or the ground-state L_n_Cu^I^–NHR species **B** to generate a cyclic iminyl radical **73a-A**, which oxidizes the L_n_Cu^II^–NHR complex **D** (Scheme 30, path a or b). Subsequently, radical **73a-A** undergoes a β-C–C bond scission to provide the cyanoalkyl radical **73a-B**, which is trapped by complex **D** and converted to the high-valent Cu^III^ complex **E**. Next, CO inserts into complex **E** to generate intermediates **F** or **G**, which undergo elimination to furnish the final product **75**. In 2020, Chen and co-worker [103] further explored the potential of this method and accomplished photoinduced the copper-catalyzed C(sp^3^)–O cross-coupling using oxime esters and phenols **76** (Scheme 30).

**Scheme 31:**
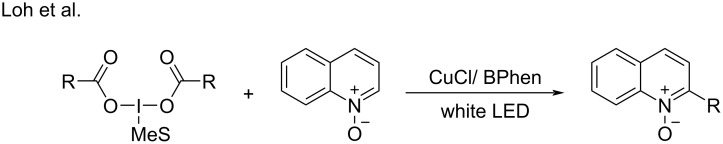
Alkylation of heteroarene *N*-oxides.

In 2020, Loh and co-workers [104] reported the copper-catalyzed highly site-selective alkylation of heteroarene *N*-oxides in the presence of hypervalent iodine^III^ carboxylates. As an alkylating agent, the hypervalent iodine^III^ carboxylates were reduced by active copper^I^ complexes and produced an alkyl radical, which was then captured by a copper^III^ active species. Finally, after reductive elimination, the target products were obtained (Scheme 31).

## Conclusion

This review highlighted the special features and applications of photoinduced copper-catalyzed reactions. Copper photoredox catalysts are powerful photocatalysts used for cross-coupling reactions. Their function is based on the strong reducing power of copper complexes and the ability of copper complexes to coordinate substrates or trap reactive intermediates. The applications of photoinduced copper-catalyzed reactions include alkene/alkyne functionalization, organic halide functionalization, and alkyl C–H functionalization. This review introduced the photoinduced copper-catalyzed stereoselective reactions within these broad reaction categories. Copper salts coordinate with diverse chiral ligands to provide a chiral environment for asymmetric control. Despite the remarkable achievements in this field, copper-based catalytic asymmetric reactions still remain a challenging task because of the difficulty of stereocontrol of the highly reactive radical intermediates. This review discussed the fundamental mechanisms underlying copper-based photocatalysis, including Cu^I^/Cu^II^-mediated and copper substrate-mediated catalytic cycles, which are important in metallo-photoredox mechanisms. The excited-state properties of Cu-based photosensitizers can be efficiently tuned by ligand modification. Although remarkable efforts have been made to elucidate and modify Cu complexes as photoredox catalysts for organic synthesis, the design of these complexes has not received much attention. If new complexes with improved redox and photophysical performances are designed, then Cu-based complexes could replace ruthenium- or iridium-based photocatalysts in the future.
